# Correction of a chronic pulmonary disease through lentiviral vector-mediated protein expression

**DOI:** 10.1016/j.omtm.2022.04.002

**Published:** 2022-04-14

**Authors:** Helena Lund-Palau, Claudia Ivette Juarez-Molina, Cuixiang Meng, Anushka Bhargava, Aikaterini Pilou, Kiran Aziz, Nora Clarke, Naoko Atsumi, Ali Ashek, Michael R. Wilson, Masao Takata, Simon Padley, Deborah R. Gill, Stephen C. Hyde, Cliff Morgan, Eric W.F.W. Alton, Uta Griesenbach

**Affiliations:** 1 National Heart and Lung Institute, Gene Therapy Group, Imperial College London, Faculty of Medicine, Manresa Road, London SW3 6LR, UK; 2 Division of Anesthetics, Pain Medicine, and Intensive Care, Department of Surgery and Cancer, Imperial College London, London, UK; 3 Royal Brompton Hospital & Harefield Hospitals, London, UK; 4 Radcliffe Department of Medicine, University of Oxford, Oxford, UK; 5 UK Respiratory Gene Therapy Consortium, London SW3 6LR, UK

**Keywords:** autoimmune pulmonary alveolar proteinosis, PAP, alveolar macrophage, surfactant protein, granulocyte-macrophage colony-stimulating factor, GM-CSF, gene therapy, lung gene therapy, rSIV.F/HN, lentiviral vector

## Abstract

We developed a novel lentiviral vector, pseudotyped with the F and HN proteins from Sendai virus (rSIV.F/HN), that produces long-lasting, high-efficiency transduction of the respiratory epithelium. Here we addressed whether this platform technology can secrete sufficient levels of a therapeutic protein into the lungs to ameliorate a fatal pulmonary disease as an example of its translational capability. Pulmonary alveolar proteinosis (PAP) results from alveolar granulocyte-macrophage colony-stimulating factor (GM-CSF) insufficiency, resulting in abnormal surfactant homeostasis and consequent ventilatory problems. Lungs of GM-CSF knockout mice were transduced with a single dose of rSIV.F/HN-expressing murine GM-CSF (mGM-CSF; 1e5–92e7 transduction units [TU]/mouse); mGM-CSF expression was dose related and persisted for at least 11 months. PAP disease biomarkers were rapidly and persistently corrected, but we noted a narrow toxicity/efficacy window. rSIV.F/HN may be a useful platform technology to deliver therapeutic proteins for lung diseases requiring long-lasting and stable expression of secreted proteins.

## Graphical abstract

**Figure:**
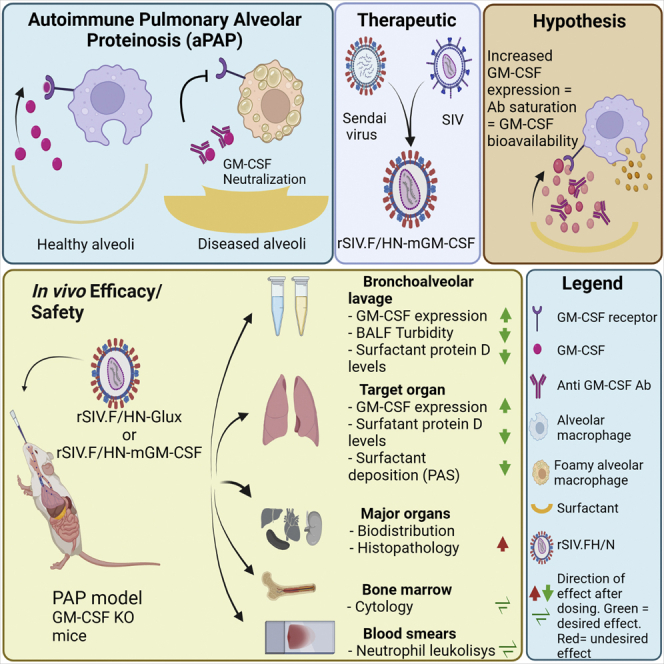

## Teaser

Efficient pulmonary gene transfer has been a limiting factor for development of gene therapy products targeting the lungs. This paper presents an efficient methodology for lung gene transfer and efficacy data for a life-limiting respiratory disease after a single dose of a novel lentiviral vector.

## Introduction

Efficient pulmonary gene transfer has been a limiting factor for development of gene therapy for chronic lung diseases such as cystic fibrosis (CF). To address this limitation, the UK Respiratory Gene Therapy Consortium (https://www.respiratorygenetherapy.org.uk) has generated a novel lentiviral vector pseudotyped with the F/HN proteins from Sendai virus (rSIV.F/HN). This vector generates high transduction efficiency in a range of lung epithelial cells, including ciliated, goblet, and club cells as well as type 1 and 2 pneumocytes; a single dose produces expression for the lifetime of a mouse, and efficient generation of proteins is maintained after three or more transductions.^1^^,^^2^ These properties suggest that it may be of therapeutic value for treatment of CF through expression of the membrane-localized CF transmembrane conductance regulator (CFTR) protein, and the vector is progressing toward a first-in-human trial.

Other lung diseases may benefit from sustained intrapulmonary expression of a secreted therapeutic protein. We have shown previously that Sendai virus can achieve this goal, albeit with only a short time course.^3^ Here we address whether the rSIV.F/HN platform technology can ameliorate an exemplary lung disease whose pathophysiology is dependent on a secreted protein. Pulmonary alveolar proteinosis (PAP) is a rare, life-limiting lung disease characterized by accumulation of surfactant in the alveoli,^4^ leading to infections, lung fibrosis,^5^ and, ultimately, respiratory failure.^6^ Surfactant homeostasis is regulated by alveolar macrophages, which catabolize excess surfactant,^7^ and granulocyte-macrophage colony-stimulating factor (GM-CSF) is required for this process.^8^^,^^9^ About 90% of PAP cases are caused by generation of anti-GM-CSF autoantibodies,^10^ which prevent adequate GM-CSF-mediated surfactant clearance by alveolar macrophages.^11^, ^12^, ^13^, ^14^ The standard of care is whole-lung lavage,^15^^,^^16^ an invasive technique where the lipoproteinaceous surfactant is washed out of each lung in turn under anesthesia at regular intervals. Inevitably, the intervention is associated with complications^17^ and is only performed in specialist centers.^6^^,^^18^ Recombinant GM-CSF protein has been administered to affected individuals subcutaneously or by aerosol to outcompete the anti-GM-CSF antibodies and, hence, restore surfactant clearance.^19^, ^20^, ^21^, ^22^, ^23^, ^24^, ^25^, ^26^ A meta-analysis indicated that such therapy may be effective and that the inhalation route may be superior.^27^ A recent double-blinded, placebo-controlled trial confirmed that daily administration of inhaled GM-CSF results in greater improvements in lung function (change in alveolar to arterial oxygen gradient, −6.2 mm Hg; p = 0.03 when comparing treated individuals and placebo recipients) and functional health status compared with placebo administration, although the changes were modest.^26^

Gene therapy for PAP may offer some advantages over recombinant protein-based treatment, including less frequent dosing, higher local GM-CSF levels, and a more stable steady-state concentration profile, which may further enhance the therapeutic index. Here we show that rSIV.F/HN carrying the GM-CSF cDNA can produce sufficient protein to correct the disease phenotype for at least 11 months in a PAP mouse model after a single administration. We suggest that these data provide a proof of concept for this core technology, allowing it to be assessed for clinical translation across a range of other lung conditions.

## Results

### rSIV.F/HN drives sustained expression of functional GM-CSF *in vitro* and *ex vivo*

To confirm that rSIV.F/HN transduction led to expression of murine GM-CSF (mGM-CSF) *in vitro*, A549 cells were transduced at a multiplicity of infection (MOI) ranging from 0.1–100. mGM-CSF was quantified 4 days after treatment. Untreated cells and cells treated with rSIV.F/HN expressing a *Gaussia* luciferase (*Glux*) reporter gene (rSIV.F/HN-*Glux*) at an MOI of 100 were included as negative controls (n = 6/group). Dose-related mGM-CSF secretion was confirmed (Figure 1A), reaching a median of 37,322 pg/mg (range, 28,865–44,784) total protein at the highest MOI (p < 0.005). *Ex vivo* transduction of fully differentiated human air-liquid interface (ALI) cultures at an MOI of ∼200 (maximum feasible dose; n = 6) led to significant (p < 0.05) and persistent expression for at least 7 months after a single dose (Figure 1B).

**Figure 1:**
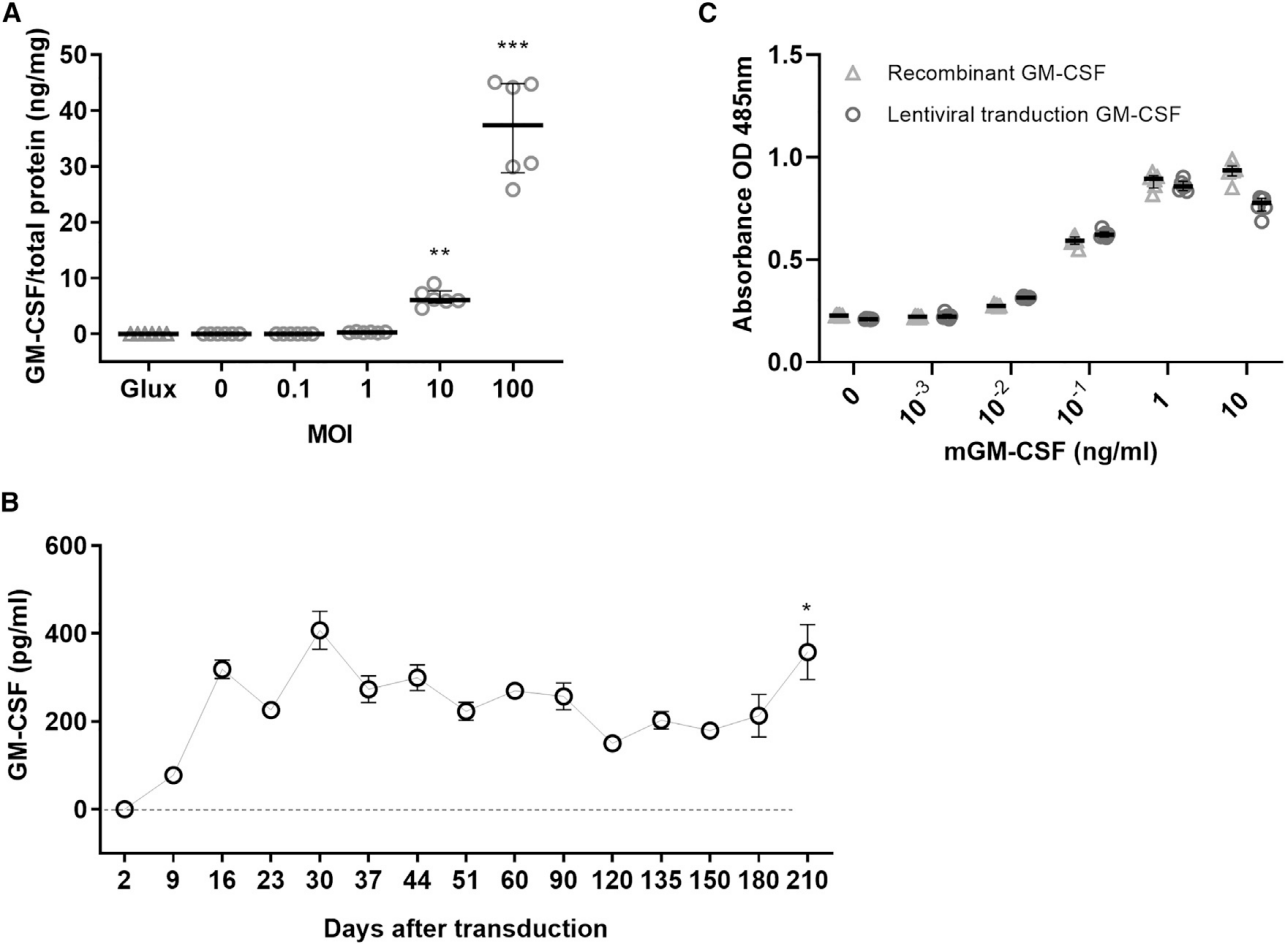
Expression of functional mGM-CSF protein after *in vitro* and *ex vivo* gene transfer (A) A549 cells were transduced with increasing multiplicity of infection (MOI; 0.1–100) of rSIV.F/HN-mGM-CSF, remained untreated (0), or were transduced with a control lentiviral vector, rSIV.F/HN-*Glux* (*Glux*) at an MOI of 100. mGM-CSF was measured in the tissue culture medium 4 days after transduction. Data are presented as median ± interquartile range (n = 6 wells/group). Kruskal-Wallis test with Dunnett correction for multiple comparisons compared with *Glux* control; ∗∗p < 0.01, ∗∗∗p < 0.005. (B) Air-liquid interface (ALI) cultures were treated at an MOI of ∼200, and the levels of secreted mGM-CSF were measured at the indicated time points. Data are presented as mean ± SEM (n = 6/group); the dotted line represents the LLD (5.8 pg/mL). One-way ANOVA with Dunn’s correction for multiple comparisons compared with the time point 2 days after transduction as the control group; ∗p < 0.05. (C) Function of mGM-CSF protein after *in vitro* gene transfer. FDC-P1 cells were exposed to known increasing concentrations of mGM-CSF from the supernatant of *in-vitro*-transduced cells and compared with commercially available recombinant mGM-CSF. These cells proliferate when exposed to GM-CSF. Cell proliferation was quantified using the One Solution cell proliferation assay and is expressed as optical density (OD) at 485 nm. Data are presented as median ± interquartile range (n = 6 well/group). ∗p < 0.05, ∗∗p < 0.01, ∗∗∗p < 0.001, ∗∗∗∗p < 0.0001.

We next assessed whether mGM-CSF, produced after lentiviral vector transduction, was fully functional using a mouse myeloid factor-dependent continuous-Paterson (FDC-P1) cell proliferation assay. FDC-P1 myeloid cells were treated with known concentrations (0–10 ng/mL) of mGM-CSF produced after *in vitro* lentiviral vector transduction and compared with equal concentrations of a commercially available recombinant mGM-CSF protein (n = 6/group). Both proteins led to similar proliferation of FDC-P1 cells (Figure 1C). These results supported the *in vivo* assessment of the lentiviral vector expressing mGM-CSF in a murine model of PAP.

### GM-CSF knockout mice recapitulate several biomarkers of human autoimmune PAP

Dranoff et al.^28^ previously described that constitutive GM-CSF knockout mice develop a PAP phenotype without an effect on hematopoiesis. Here we first confirmed changes in disease biomarkers, including significant increases in bronchoalveolar lavage fluid (BALF) turbidity, surfactant protein D (SP-D) levels in BALF and lung tissue homogenate, increased surfactant deposition of periodic acid-Schiff (PAS)-positive material in lung tissue, and enlarged alveolar macrophages (Figures S1A–S1F), compared with age-matched wild-type mice (n = 8–36/group). There was no correlation between disease biomarkers and murine age, except for deposition of PAS-positive material in alveoli and SP-D levels in BALF (Figures S1G and S1H).

We next addressed whether imaging or physiological measurements, cornerstones of disease monitoring in humans, might also be applicable to this murine model. Computed tomography (CT) scans were performed for knockout mice and age-matched littermates (n = 6/group). Although we observed a small but significant (p < 0.01) increase in lung density in GM-CSF knockout mice (Figure S1I), power calculations indicated that the modest differences precluded use of this biomarker in further studies. We also compared, for the first time, the alveolar-arterial (A-a) gradient and partial pressure of oxygen (PaO_2_). Respiratory mechanics, including elastance and pressure-volume (PV) curves, were also described for the first time. Differences in lung compliance, measured as the change in lung volume and pressure (PV curves), were not detected when comparing knockout and wild-type mice (Figure S1J). Although a small reduction in PaO_2_ and increase in A-a gradient were seen in knockout animals compared with wild-type littermates, neither reached significance (Figures S1K and S1L). A longitudinal study assessing lung disease with increasing age may help to refine the role, if any, of these biomarkers in this murine PAP model.

### Transduction with rSIV.F/HN-mGM-CSF leads to dose-related mGM-CSF expression and correction of PAP biomarkers

The lungs of PAP mice were treated with rSIV.F/HN-mGM-CSF via intranasal sniffing at doses ranging from 1e5 transduction units (TU)/mouse to the maximum feasible dose of 92e7 TU/mouse (n = 2–18/group) or with the irrelevant control vector (rSIV.F/HN-*Glux*, n = 36) at the maximum feasible dose of 24e7 TU/mouse. The maximum feasible dose was defined as the maximum amount of vector (based on the vector concentration) that could be delivered in a 100-μL volume, the maximal allowable volume based on Home Office regulations.

mGM-CSF expression was quantified in BALF, lung homogenate, and serum 2 months after vector administration. Dose-related mGM-CSF expression (p < 0.005) was detected in BALF and lung homogenates in mice treated with ≥ 1e7 TU/mouse (Figures 2A and 2B). mGM-CSF was also detectable in serum in the two highest dosing cohorts (Figure 2C), indicating release of GM-CSF into the circulation, which may contribute to the observed histopathological changes (see below) and may be consistent with the lack of detectable protein in wild-type animals. In contrast, mGM-CSF was undetectable in untreated wild-type or GM-CSF knockout mice treated with the control *Glux* vector (Figures 2A and 2B).

**Figure 2:**
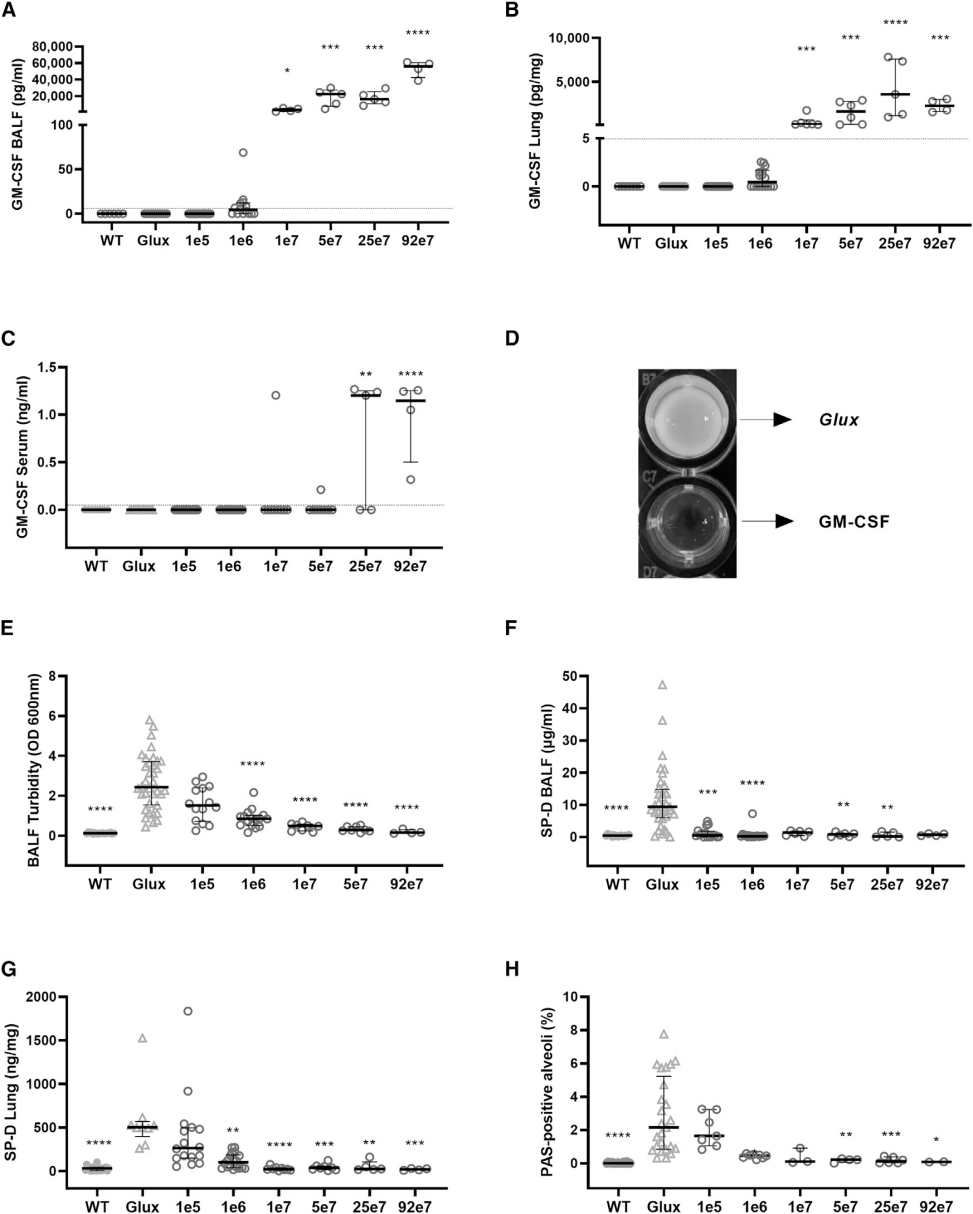
Dose-related mGM-CSF expression ameliorates biomarkers of PAP disease Lungs of GM-CSF knockout mice were treated with increasing doses of the rSIV.F/HN-mGM-CSF vector (1e5–92e7 TU/mouse) or the *Glux* control vector. Untreated wild-type (WT) mice are included for reference. mGM-CSF expression was quantified 2 months after treatment in (A) bronchoalveolar lavage fluid (BALF), (B) lung homogenate, and (C) serum. The effect of mGM-CSF expression on biomarkers of PAP were analyzed: (D) representative image of BALF turbidity from an animal treated with 1e7 TU of rSIV.F/HN-mGM-CSF or control *Glux* lentiviral vector, (E) BALF turbidity measured by absorbance, (F) surfactant protein D (SP-D) concentration in BALF, (G) SP-D concentration in lung homogenate, (H) surfactant deposition in the alveoli quantified as percentage of PAS-positive alveoli. Data are presented as median ± interquartile range (n = 2–36/group); a dotted line represents the LLD (5.8 pg/mL for mGM-CSF and 9.4 pg/mL for SP-D). Kruskal-Wallis test with Dunnett correction for multiple comparisons compared with the *Glux* control; ∗p < 0.05, ∗∗p < 0.01, ∗∗∗p < 0.005, ∗∗∗∗p < 0.001.

We next assessed whether administration of a lentiviral vector expressing an irrelevant reporter gene (*Glux*) had any effect on the selected pulmonary biomarkers prior to embarking on a larger study. No significant changes were observed, suggesting that rSIV.F/HN-mGM-CSF was an appropriate vector to assess for therapeutic benefit (Figures S2A–S2D).

Subsequently, mice were transduced with rSIV.F/HN-mGM-CSF at doses ranging from 1e5–92e7 TU/mouse (n = 7–36/group) and compared with rSIV.F/HN-*Glux* treated controls 2 months after vector administration. BALF turbidity, SP-D levels in lung homogenate, and surfactant deposition in alveoli (PAS staining) were reduced significantly (p ≤ 0.05–0.001) in rSIV.F/HN-mGM-CSF-treated PAP mice treated with 1e6 TU/mouse or greater, and SP-D levels in BALF were reduced significantly (p ≤ 0.01) with 1e5 TU/mouse or greater (Figures 2D–2H).

### Transduction with rSIV.F/HN-mGM-CSF leads to persistent mGM-CSF expression and long-lasting amelioration of PAP biomarkers

To determine the duration of mGM-CSF expression and amelioration of PAP biomarkers after a single dose, PAP mice were treated with rSIV.F/HN-mGM-CSF or the control vector rSIV.F/HN-*Glux* (1e7 TU/mouse) and culled 1 week to 9 months after administration (n = 3–8/time point). mGM-CSF was detectable (p < 0.001) in lung tissue for the duration of the study (9 months) and in BALF for ∼2 months after transduction (Figures 3A and 3B). Levels were only detectable in serum at the 1-week time point in 3 of 8 mice (data not shown).

**Figure 3:**
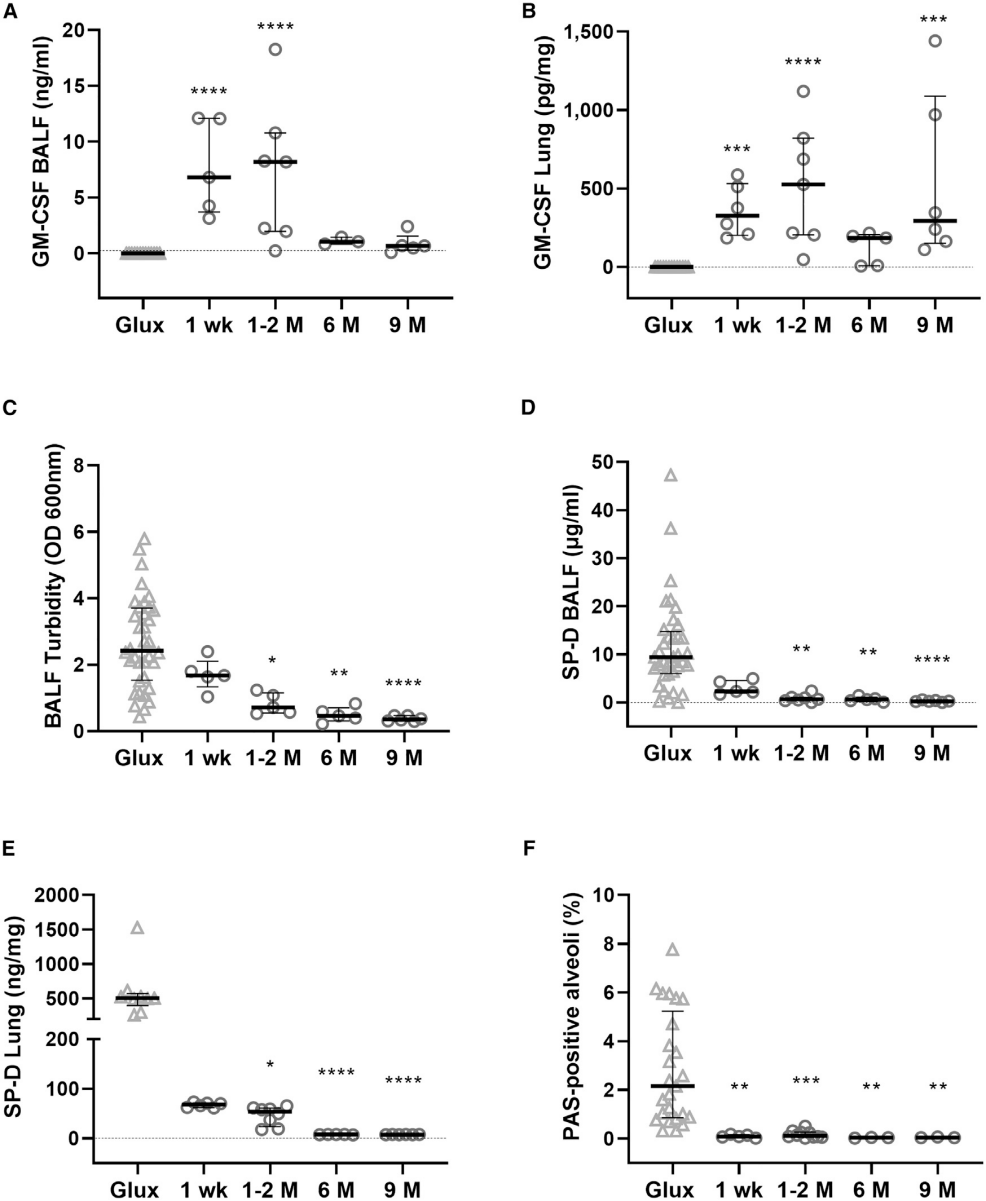
Sustained treatment effect after a single dose of rSIV.F/HN-mGM-CSF Lungs of GM-CSF knockout mice were treated with the rSIV.F/HN-mGM-CSF lentiviral vector (n = 3–9/group) or the *Glux* control vector (1e7 TU/mouse) (n = 13–36/group). Animals were culled 1 week to 9 months after transduction, and mGM-CSF expression was quantified in (A) BALF and (B) lung homogenate. In addition, the long-term effect of mGM-CSF expression on biomarkers of PAP disease were analyzed: (C) BALF turbidity, (D) SP-D concentration in BALF, (E) SPD concentration in lung homogenate, and (F) surfactant deposition in alveoli quantified as percentage of PAS-positive alveoli. Data are presented as median ± interquartile range (n = 3–8/group); a dotted line represents the LLD (5.8 pg/mL for mGM-CSF and 9.4 pg/mL for SP-D). Kruskal-Wallis test with Dunnett’s correction for multiple comparisons compared with the *Glux* control group. ∗p < 0.05, ∗∗p < 0.01, ∗∗∗p < 0.005, ∗∗∗∗p < 0.001.

In keeping with prolonged expression of mGM-CSF, PAP disease markers, including BALF turbidity, SP-D levels in BALF and lung tissue homogenate, and surfactant deposition in the alveoli, were reduced significantly (p < 0.05) as early as 1 week after transduction and remained significantly reduced (p ≤ 0.05) for the duration of the study (9 months after treatment) after a single administration of the vector (Figures 3C–3F).

### Defining an efficacy/toxicity window for rSIV.F/HN mGM-CSF expression in GM-CSF knockout mice

GM-CSF has several functions within the endogenous immune/inflammatory cascade, including differentiation of granulocytes (neutrophils, eosinophils, and basophils) and monocytes/macrophages from precursors;^28^ it appears to be a tightly regulated protein and is therefore likely to have a well-defined efficacy/toxicity window.

In an initial dose-finding study, mice were treated with 1e7–92e7 TU/mouse. Figure S3A shows a blinded, semi-quantitative histological assessment of the major organs (lungs, liver, kidneys, spleen, heart, skeletal muscle, gonads, and thymus) 2 months after vector administration. Untreated PAP mice showed mild pulmonary inflammatory changes and evidence of alveolar thickening as well as a mild phenotype in the liver, both likely related to the lack of GM-CSF expression in these knockout mice.

Transduction with the *Glux*-expressing control vector did not alter these baselines changes. Administration of rSIV.F/HN-mGM-CSF (1e7–92e7 TU/mouse, n = 3–6/group) led to dose-related histopathological changes in the lungs, liver, and kidneys (Figure S3A), which are likely to have contributed to some mice in the two highest dose cohorts having unexpected deaths (3 of 11 and 5 of 12 for the 24e7 and 92e7 TU/mouse cohorts, respectively). In addition, spleen size increased in a dose-related manner (Figures S3B and S3C) without altering histology (Figure S3A). We did not observe histopathological changes in the heart, skeletal muscle, gonads, or thymus at any dose (data not shown). In animals treated with 1e5 and 1e6 TU/mouse, we saw no toxicity, based on histological changes or mortality 2 months after treatment (data not shown).

Given the prolonged expression characteristic of the rSIV.F/HN platform technology, we next assessed whether longer-term expression of mGM-CSF in mice receiving 1e7 TU/mouse would lead to histopathological changes. Animals were treated with rSIV.F/HN-mGM-CSF or the *Glux* control vector and followed up for 9 months after treatment (n = 3–10/time point), with histological assessment performed as described above. In the former group, we observed progressively more severe histopathological changes in the lungs 6–9 months after treatment, including tissue consolidation, inflammation, and increased numbers of pulmonary macrophages (Table S1). In addition, at the 9-month time point, we also saw an accumulation of inflammatory cells and blood vessel dilation in the kidneys. Analyses of all other organs, including blood smears, BALF cytospins, and bone marrow, were unremarkable (data not shown).

To further define a suitable efficacy/toxicity window in this model, we extended the histopathological analysis to an 11-month time point in animals treated with 1e5 and 1e6 TU/mouse (n = 4–7/group). As noted above, mice treated with these comparatively low doses showed significant improvements in PAP biomarkers even though levels of vector-induced mGM-CSF levels were below the lower limit of detection for the assay. Histopathological analysis at this time point showed no or moderate histopathology at 1e5 or 1e6 TU/mouse, respectively (Table 1). We therefore were able to define a window of efficacy/toxicity in this murine model. Likely related to the long duration of expression produced by the rSIV.F/HN platform, we saw evidence of a cumulative effect of GM-CSF with time, allowing lower titers of the rSIV.F/HN vector to be administered.

**Table 1:** Histopathological changes after pulmonary administration of ultra-low doses of rSIV.F/HN-mGM-CSF to GM-CSF knockout mice

	*Glux*	1e5	1e6
**Lungs**			
Distorted architecture	–	–	–
Inflammatory cell infiltration	++	++	+++
Alveolar wall thickness	++	–	–
PAM alveoli	–	–	++
PAM bronchi	–	−/+	−/+
Neutrophil bronchi	–	–	−/+
Consolidation	–	–	–
Giant cells	–	–	–
Eosinophilic material	–	–	–
Edema	–	–	–
**Liver**			
Inflammatory cell infiltration	++	–	+++
Portal area inflammation	–	–	++
Dilated congested sinusoids	++	–	–
Dilated congested blood vessels	–	–	−/+
**Kidneys**			
Inflammatory cell infiltration	++	++	+++
Dilated blood vessels	−/+	–	++
Fibrosis	–	–	–
Eosinophilic material	−/+	−/+	++
Cysts	–	–	−/+
**Spleen**			
Clusters of megakaryocytes	–	–	–
Macrophages	–	–	–

Lungs of GM-CSF knockout mice were treated with the rSIV.F/HN-mGM-CSF vector (1e5 or 1e6 TU/mouse) or the *Glux* control lentiviral vector (2.4e9 TU/mouse) (n = 4–7 mice/group). 11 months after treatment, analysis was performed blinded using a semi-quantitative scoring system. Scores are defined as follows: −, absent; −/+, equivocal; ++, mild; +++, moderate; ++++, severe. PAM, pulmonary alveolar macrophage.

## Discussion

Here we assess the feasibility of using a novel platform technology, rSIV.F/HN, to generate long-lasting protein secretion within murine lungs. As a therapeutic example, efficacy and toxicity were studied in a model of PAP, a disease with a considerable unmet need. mGM-CSF expression in murine lungs was dose related and persisted for the duration of the 11-month study after a single administration. Disease biomarkers were rapidly and persistently corrected. A narrow efficacy/toxicity window could be defined, with only low doses needing to be administered for therapeutic benefit, likely related to the long-lived mGM-CSF expression.

There is a considerable unmet need across a broad swathe of respiratory diseases of genetic and of multifactorial etiology; many of these are chronic and likely require sustained levels of therapeutic proteins. Recombinant proteins require frequent administration, are often associated with difficulties in achieving therapeutic levels at the required site of action, and are expensive. Thus, daily administration of inhaled GM-CSF resulted in modest improvements in pulmonary gas transfer and functional health status compared with placebo treatment in individuals with PAP;^26^ symptoms reappeared approximately 5 weeks after cessation of treatment.^29^ Nucleic acid-based technologies offer a theoretical solution, but, to date, progress has been slow. We and others have shown that, in contrast to adenoviral and adeno-associated virus-based vectors, lentiviral vectors can be repeatedly administered without losing efficacy^30^^,^^31^ and, hence, are well suited for treatment of chronic lung diseases. We have shown previously in mice that the rSIV.F/HN vector efficiently transduces ciliated, goblet, and club cells as well as type 1 and 2 pneumocytes.^2^ We do not foresee a difference in transduction profile in GM-CSF knockout mice, and considering that GM-CSF is a secreted protein, the transduced cell type is not expected to have an effect on the efficacy of the treatment. Finally, a single administration results in expression for the lifetime of a mouse. We therefore sought evidence that this platform technology could produce a therapeutic functional protein to ameliorate an exemplary human disease modeled in the mouse.

Reed et al.^29^ have shown previously that administration of recombinant GM-CSF reverses the PAP phenotype in GM-CSF knockout mice. In addition, Huffman et al.^8^ have demonstrated that transgenic mice expressing GM-CSF under the control of a SP-C promoter show correction of the PAP phenotype. These studies validate the role of GM-CSF as a key therapeutic protein in this disease and provide a helpful comparator against which to benchmark a nucleic acid-based treatment strategy. Zsengeller et al.^32^ showed that administration of an adenoviral vector expressing GM-CSF leads to transient (1–3 weeks) low levels of GM-CSF expression, which corrects some biomarkers of the disease. However, it has been well documented that adenoviral vectors induce immune responses, lose efficacy when administered repeatedly,^33^ and are therefore unlikely to be suitable for treatment of chronic lung diseases.

We were readily able to achieve therapeutic GM-CSF doses over prolonged periods with our lentiviral vector. Above the relatively low titer of 1e7 TU/mouse, no further increases in pulmonary GM-CSF production or disease amelioration were seen. Although these data were encouraging at the 2-month time point, as the mice aged, we observed histopathology within and outside of the target organ. The histopathological changes observed were consistent with the known biological functions of mGM-CSF: differentiation of granulocytes and monocytes/macrophages from progenitor cells^34^ and enhancement of neutrophil migration.^35^ The observed histopathology changes are also consistent with previous reports showing that exposure of mice to high levels of mGM-CSF causes significant histopathological changes.^36^^,^^37^ Finally, rare case studies in humans have described splenic enlargement^38^ and rupture after recombinant GM-CSF treatment.^39^

Therefore, we next assessed the effect of lower viral titers. mGM-CSF levels were below the lower limit of detection (LLD) of the ELISA in BALF, lung homogenate, and serum in animals dosed with 1e6 TU/mouse, but a clear phenotypic effect could be seen. This is consistent with the fact that mGM-CSF levels in wild-type mice are also below the LLD for the ELISA, indicating that low levels of mGM-CSF produced *in situ* are required to stimulate macrophages to regulate surfactant levels. As the titer was reduced below 1e7, we could demonstrate a narrow efficacy/toxicity window and also noted a cumulative temporal effect of sustained GM-CSF production. These data suggest that only low titers of the rSIV.F/HN platform might be needed to generate therapeutic levels at the required site of action.

This study was not focused on therapeutic translation, but because of the relatively narrow efficacy/toxicity window in mice, we took the opportunity to further refine the lentiviral vector cassette by removing its integration capability into the target cell genome (integration-deficient lentiviral vector), leading to more transient expression of GM-CSF or the option of using weaker or regulated promoters. However, a key element relates to whether efficacy and toxicity are altered in the presence of anti-GM-CSF autoantibodies, as is the case in man. Feretti et al.^40^ characterized Rasgrp1-deficient mice, which develop autoantibody-mediated PAP. The disease phenotype manifests only in older mice (older than 6 months) and is associated with high mortality, which makes this model difficult to work with. Sakagami et al.^13^^,^^41^ have shown that prolonged exposure of non-human primates to anti-GM-CSF antibodies can induce a PAP-like phenotype. However, this model is complex and does not lend itself to early proof-of-concept studies. Passive immunization of mice with anti-mGM-CSF antibodies might provide an alternative model. We have identified two hybridoma cell lines that produce murine anti-GM-CSF antibodies^42^ and are assessing whether passive immunization of wild-type mice with these antibodies induces a PAP-like phenotype. If successful, this model may be a viable option to assess the next steps in the PAP-specific translational pathway.

Here we have shown a proof of concept that a single administration of a lentiviral vector encoding a secreted protein can reverse the lung phenotype of a chronic respiratory disease. This may provide a useful platform technology for other chronic lung diseases requiring long-lasting exposure to a secreted therapeutic protein.

## Methods

### Lentiviral production

rSIV.F/HN carrying mGM-CSF (GenBank: AY950559) or *Gaussia* luciferase (*Glux*) cDNA under control of a hybrid elongation factor 1α promoter/Cytomegalovirus (CMV) enhancer were produced as described previously.^1^^,^^31^

### *In vitro* and *ex vivo* transduction

Human adenocarcinoma alveolar basal epithelial cells (A549, CCL-185, American Type Culture Collection, VA, USA) were cultured in complete medium (Dulbecco’s modified Eagle’s medium, 10% fetal bovine serum, 1% penicillin/streptomycin) and transduced in Opti-MEM (Fisher Scientific, Loughborough, UK), with (1) rSIV.F/HN-mGM-CSF, (2) the control lentiviral vector rSIV.F/HN-*Glux*, or TSSM diluent buffer (20 mM tromethamine, 100 mM NaCl, 10 mg/mL sucrose, and 10 mg/mL mannitol). mGM-CSF levels were quantified in medium using an mGM-CSF-specific ELISA (R&D Systems, Abingdon, UK) according to the manufacturer’s recommendations. The total protein concentration per well was quantified using cell lysates and Bio-Rad DC Protein Assay reagents (Bio-Rad, Hertfordshire, UK). GM-CSF expression was expressed as picograms of mGM-CSF per milligram of total protein.

Fully differentiated human air-liquid interface (ALI) cultures were purchased from Epithelix (Geneva, Switzerland). ALIs were transfected with rSIV.F/HN-mGM-CSF (MOI, ∼200) for 4 h in Opti-MEM or diluent control. Epithelial lining fluid was collected every 2–3 days by washing the apical surface of the ALIs with 100 μL PBS, and mGM-CSF levels were quantified by ELISA.

### GM-CSF bioassay

FDC-P1 cells (murine myeloid cells, CRL-12103, American Type Culture Collection, VA, USA) were exposed to increasing concentrations of mGM-CSF generated *in vitro* by rSIV.F/HN-mGM-CSF (0.001–10 ng/mL), and values were compared with cells exposed to recombinant mGM-CSF (PeproTech, London, UK). The “One Solution” cell proliferation assay (Promega) was used to estimate cell numbers according to the manufacturer’s recommendation.

### *In vivo* transduction of murine lungs

All animal studies were approved by the Imperial College Animal Ethics Committee and carried out according to Home Office regulations. Wild-type (C57BL/6N, Charles River Laboratories, Margate, UK) and GM-CSF knockout (B6.129S-Csf2^tm1Mlg^/J, The Jackson Laboratory, Bar Harbor, Maine, USA) mice were used. Ages at the time of analysis ranged from 2–12 months, and treatment groups were generally aged matched. Mice were anesthetized with isoflurane (200 mg/mL, FDG9623, Baxter, Norfolk, UK) and transduced with rSIV.F/HN carrying mGM-CSF or the *Glux* reporter gene through nasal instillation of 100 μL total volume, which was then rapidly sniffed into the respiratory tract.

At the indicated time points, mice were culled through intraperitoneal injection of pentobarbital (pentobarbitone sodium 20%, Animalcare, York, UK) and exsanguinated by femoral transection. Blood was collected from the heart, and whole-blood smears were prepared using standard procedures. The remaining blood was centrifuged at ∼7,200 × *g* for 15 min to extract the serum. Bronchoalveolar lavage was performed using 0.5 mL PBS (pH 7.4). Lung tissue was snap frozen in dry ice or fixed in 4% paraformaldehyde (PFA) for subsequent histopathological analysis. Other major organs were also collected and snap frozen for ELISAs and phosphatidylcholine (PC) and protein assays or fixed in 4% PFA for histopathological analysis. Bone marrow samples were isolated using a flushing technique, spun into cytospins (∼20,000 cells/slide), and the remaining cells were stored at −80°C in 10% dimethyl sulfoxide (DMSO) in fetal bovine serum (FBS).

### BALF turbidity and macrophage morphology

BALF turbidity was analyzed at 600-nm absorbance (FLUOstar Omega microplate reader, BMG Labtech, Aylesbury, UK). Cells in BALF and from bone marrow were cytospun onto slides (∼20,000 cells/slide) and fixed with ice-cold methanol (Fisher Scientific, Loughborough, UK) for 5 min and left to air dry at room temperature. Slides were then stained with oil red O using standard procedures. Images (10/slide) were captured with an inverted light microscope (20×, Telaval 31 inverted microscope, Carl Zeiss, Cambridge, UK). Using FIJI-ImageJ2 software, the size of wild-type and GM-CSF knockout mouse macrophages was measured (∼50 and 70 cells for wild-type and knockout mice, respectively).

### SPD and GM-CSF quantification

Snap-frozen lung tissue samples were thawed and homogenized using RNase/DNase-free Lysing Matrix D tubes (Fisher Scientific, Loughborough, UK) and cell lysis buffer 2 (R&D Systems, Abingdon, UK). Homogenized tissue was filtered through a QIAshredder disposable cell lysate homogenizer (QIAGEN, Manchester, UK), and the supernatant was stored at −80°C. Total protein concentration of the lung homogenate was quantified using the Bio-Rad DC Protein Assay. The concentration of SP-D in BALF and lung homogenate was determined using the Quantikine ELISA Mouse SP-D Kit (MSFPD0, R&D Systems, Abingdon, UK), following the manufacturer’s instructions. The concentration of mGM-CSF was determined using the Quantikine ELISA Mouse GM-CSF Kit, following the manufacturer’s instructions.

### Lung CT scans and oxygenation parameters

This information is supplied in the supplemental information.

### Histopathology

PFA-fixed lungs were embedded in paraffin, sectioned, and stained with hematoxylin and eosin (H&E) using standard procedures. A blinded, semi-quantitative analysis was undertaken. PAS staining was performed according to standard procedures to quantify surfactant deposition in the lungs. Images were captured and analyzed using ImageJ software, utilizing the color deconvolution macro (algorithm *H PAS 2*) for automatic quantification of PAS-positive areas based on color intensity. Briefly, each montaged whole-lung coronal cross-section underwent color deconvolution to isolate only PAS-positive areas, which were enumerated and normalized against the total lung area to give a PAS percentage area readout. Surfactant deposition was defined as the percentage of PAS-positive area of the total lung area analyzed. In addition, other major organs were fixed in PFA, cut, and stained with H&E using standard procedures to quantify histopathological changes.

### Statistical analysis

The data originated from 7 individual experiments; each experiment included a concurrent irrelevant vector control group (rSIV.F/HN-*Glux*). Given that there were no statistical differences between the control groups in the different experiments, data were combined as a historic control cohort. Except where specified, data are presented as median with interquartile range. Statistical analysis was performed using Mann-Whitney or Kruskal-Wallis test with Dunn’s correction for multiple comparisons, as appropriate. The null hypothesis was rejected at p < 0.05. Statistical analysis was performed using GraphPad Prism 8 software.

## Supplemental information

Document S1 Figures S1–S3 and Table S1

Document S2. Article plus supplemental information
