# Supplementary material for: Correction of a chronic pulmonary disease through lentiviral vector-mediated protein expression
Source: Mol Ther Methods Clin Dev. 2022 Apr 14;25:382–91. doi: 10.1016/j.omtm.2022.04.002 (PMC9065048; doi:10.1016/j.omtm.2022.04.002)
Supplement: Document S1 Figures S1–S3 and Table S1 [file mmc1.pdf]

OMTM, Volume *25*

Supplemental information

Correction of a chronic pulmonary disease through lentiviral vector-mediated protein expression

Helena Lund-Palau, Claudia Ivette Juarez-Molina, Cuixiang Meng, Anushka Bhargava, Aikaterini Pilou, Kiran Aziz, Nora Clarke, Naoko Atsumi, Ali Ashek, Michael R. Wilson, Masao Takata, Simon Padley, Deborah R. Gill, Stephen C. Hyde, Cliff Morgan, Eric W.F.W. Alton, and Uta Griesenbach

Lung computed tomography (CT) scans

CT imaging experiments were conducted at the Biological Imaging Centre, Imperial College London, using the Siemens Inveon PET/CT (Siemens Medical Solutions USA, Inc). Mice were anesthetized using isoflurane (5% for induction and 1.5% for maintenance in 100% oxygen with a flow rate of 1.5 L/min). The animals were placed into the CT scanner on the mouse-bed equipped with a nose cone, respiratory pillow (M2M, USA) and electrical heating pad (37ºC). Finally, the animal was centred in the CT field of view using laser alignment to cover the whole lung. Respiration gated CT acquisition was performed using 180 projections over the 360 degrees rotation. Each projection lasts for 200 ms of exposure time with 80V tube voltage and 500 µA tube current. X-ray detector binning was set to 2, which conferred an isotropic spatial resolution of 34.61 µm.

To reduce the radiation exposure to the animals and facilitate follow-up scanning, the scanner mode was converted from conventional helical scanning to step and shoot mode with a settle time of 50 ms (this conversion also limited the vibration induced by the rotation of the gantry, hence minimising motion blurring artefacts). The scan time for each animal was approximately 5 minutes. Body temperature and respiration rates were continuously monitored during the scan using BioVet physiological monitoring system (m2m Imaging Corp, Cleveland OH). The CT scan projections were reconstructed using Feldkamp algorithm using Shepp Logan filter with no downsampling. Images were analysed by using Inveon Research Workplace 1.4 (Siemens Medical Solutions USA, Inc.) Regions of interest were drawn over the whole lung and further refinement was performed using CT density thresholding. Mean lung density of the whole lung in Hounsfield units (HU) was compared between wildtype and PAP mice.

Lung physiology parameters

Mice were anaesthetised by intraperitoneal administration of ketamine (80 mg/kg) and xylazine (8 mg/kg). An endotracheal tube was placed through tracheostomy and mice were connected to a custom-made ventilator/pulmonary function testing system. The carotid artery was cannulated for continuous infusion of heparinised (10 U/ml) saline solution (0.3 ml/hour), monitoring of arterial blood pressure and removal of samples for blood gas analysis. Body temperature was monitored via a rectal temperature probe and maintained by an external heat source. As soon as mice were placed onto the ventilator their lungs were recruited by sustained inflation (30 cm H_2O for 5 seconds) and animals were thereafter ventilated with a tidal volume of 8-9 ml/kg using 100% O_2, positive end-expiratory pressure of 3 cm H_2O, respiratory rate of 120 breaths per minute, for 20 minutes. The blood sample collected at the end of the 20 minutes provided the arterial O_2 (PaO_2).

Respiratory system elastance was then determined by the end-inflation occlusion technique. The Alveolar-arterial gradient (A-a gradient) was calculated from the arterial blood sample using the alveolar gas equation, with the following assumptions i) barometric pressure of 760 mm Hg; ii) water vapour pressure of 0, as pressurised cylinders containing dry gas were used for ventilation; iii) FiO_2 of 1.0 (100% O_2); and iv) a respiratory quotient of 0.9.

The pressure-volume (PV) curves were performed after other measurements were completed. These were generated by gradually increasing the tidal volume during mechanical ventilation up to total lung capacity and then decreasing back to ‘normal’ and plotting plateau pressure (during end inflation occlusion) against delivered tidal volume.

Supplementary Figure 1: Disease biomarker characterization

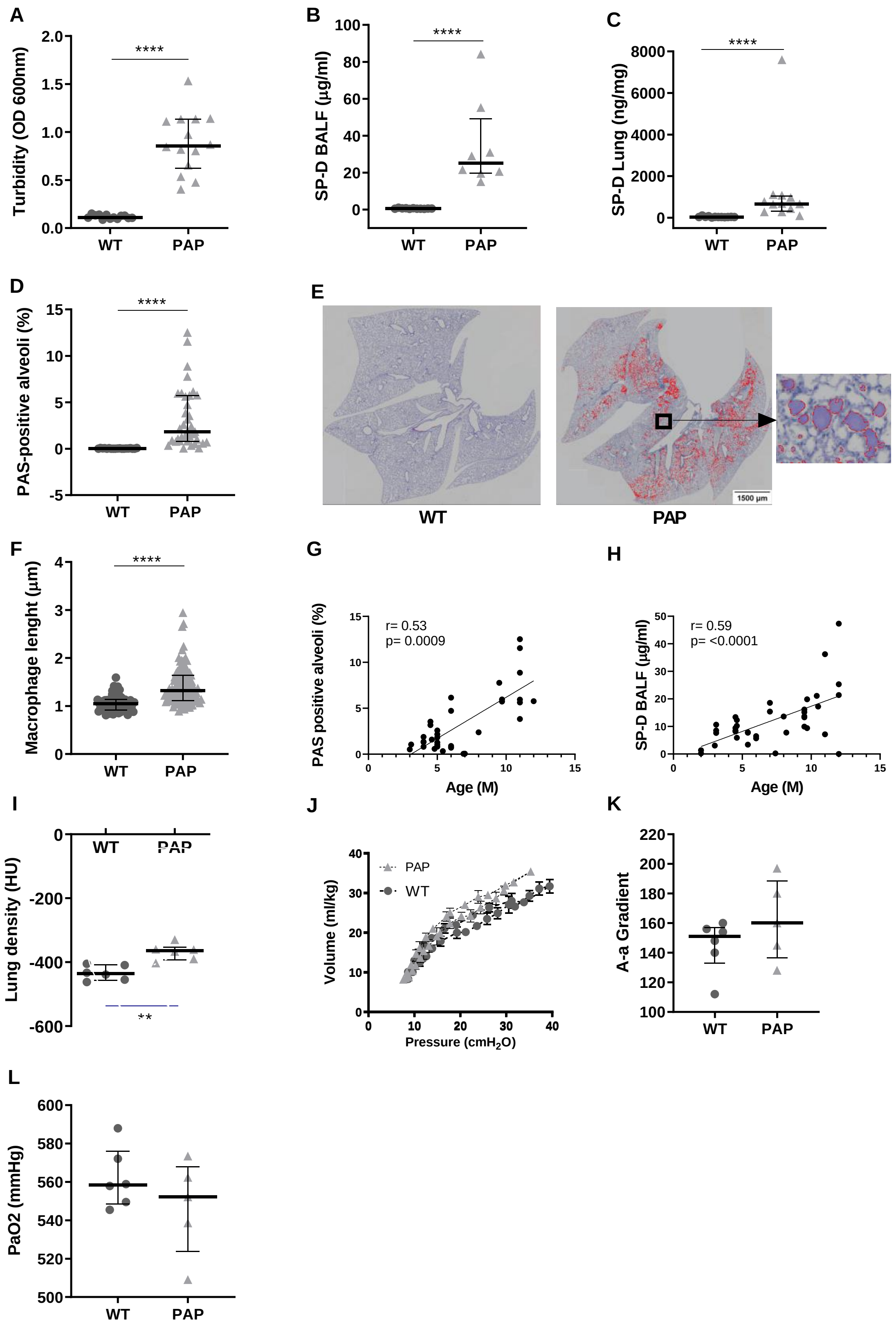

Age-matched GM-CSF knockout mice (PAP) and wildtype (WT) mice were compared for **(A)** BALF turbidity, **(B)** SP-D concentration in BALF and **(C)** SPD concentration in lung homogenate. **(D)** Percentage of PAS-positive alveoli, **(E)** Representative images of PAS-positive material (in red) in the alveoli of wildtype (WT) and GM-CSF knockout (PAP) mice, images were captured at 10x magnification, scale bar 1,500 µm. **(F)** Alveolar macrophage size. **(A-F)** Data are presented as median ± interquartile range (n=8-36 per group), Mann-Whitney test **** = p<0.0001. **(G)** Percentage of PAS-positive alveoli and **(H)** SP-D concentration in BALF positively correlate with age in GM-CSF knockout mice, two-tailed Spearman correlation (n= 41 per group). **(I)** Lung tissue density quantified from CT scans. **(L)** Pressure-volume (PV) curves measured by increasing and decreasing the tidal volume as described in the methods (n=5/group). **(M)** Oxygenation parameters were also compared (n=5-6/group) by measuring Alveolar-arterial gradient (A-a gradient) and **(N)** partial pressure of oxygen (PaO_2). **(K-N)** Data are presented as median ± interquartile range, Mann-Whitney test ** =p<0.01.

Supplementary Figure 2: Comparison of biomarkers in untreated and GM-CSF knockout mice treated with the *Glux* control vector

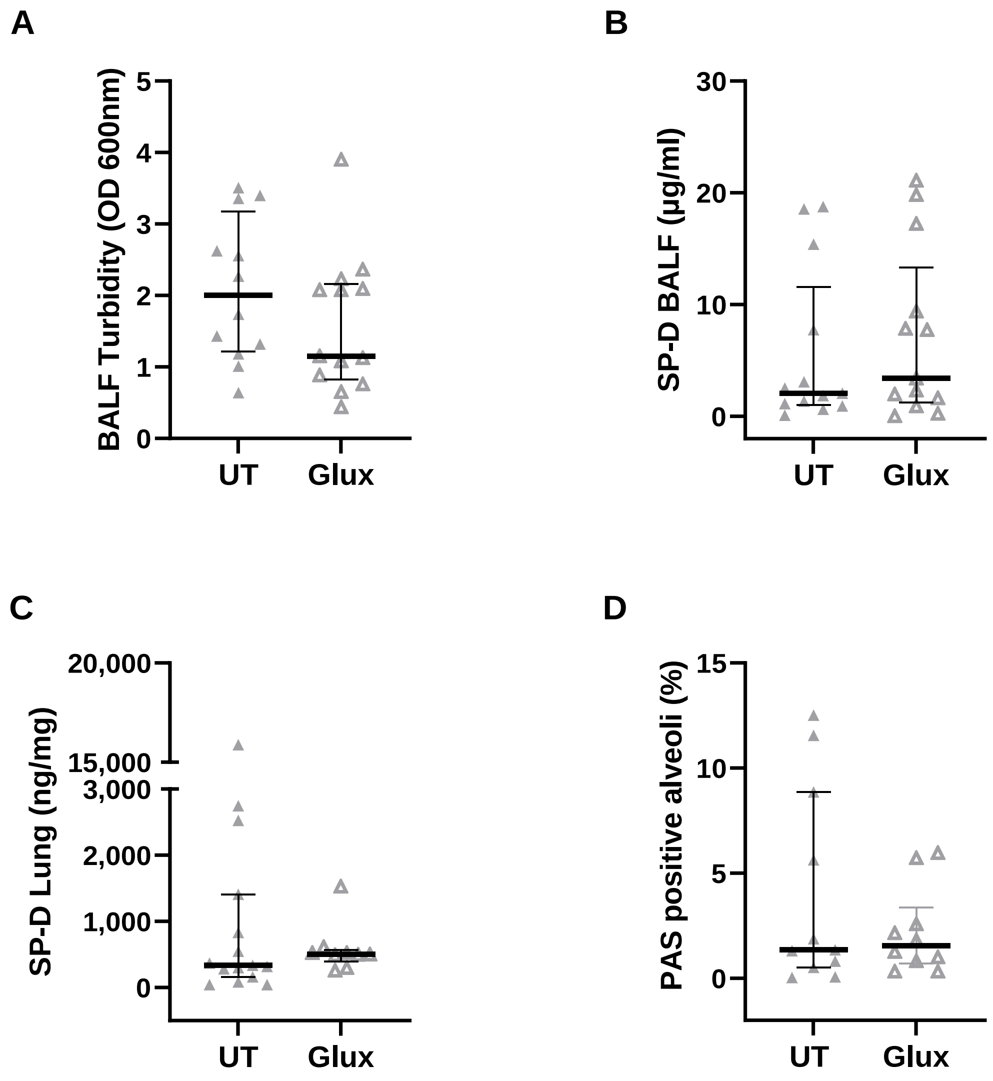

Lungs of GM-CSF knockout mice were treated with a control lentivirus (24e7 TU/mouse) expressing a *Gaussia luciferase* (*Glux*) reporter gene and disease biomarkers were compared with untreated (UT) knockout mice 2-3 months post-transduction (n=9-13/group). **(A)** Bronchoalveolar lavage fluid (BALF) turbidity measured by absorbance, **(B)** surfactant protein D (SP-D) concentration in BALF and **(C)** SPD concentration in lung homogenate. **(D)** Surfactant deposition in the alveoli was quantified as the percentage of PAS-positive alveoli. Data are presented as median ± interquartile range, Mann-Whitney test.

Supplementary Figure 3: Dose-related histopathological changes after pulmonary administration of rSIV.F/HN-mGM-CSF to GM-CSF knockout mice

A

	WT	PAP	*Glux*	1e7	5e7	25e7	92e7
**Lung**							
Distorted architecture	-	-	-	-	-	+++	++++
Inflammatory cell infiltration	-	++	++	+++	+++	++++	++++
Alveolar wall thickness	-	++	++	++	+++	++++	++++
PAM alveoli	-	-	-	++	+++	++++	++++
PAM bronchi	-	-	-	-	++	+++	++++
Neutrophils bronchi	-	-	-	-	-/+	+++	++++
Consolidation	-	-	-	++	++	++++	++++
Giant cells	-	-	-	++	-/+	+++	++++
Eosinophilic material	-	-	-/+	-	-/+	-/+	+++
Oedema	-	-/+	-	-	-	-/+	+++
**Liver**							
Inflammatory cell infiltration	-	++	++	++	+++	++++	+++
Portal area inflammation	-	-/+	-	-	-/+	++++	++
Dilated congested sinusoids	-	-/+	++	-	-/+	+++	+++
Dilated congested blood vessels	-	-/+	-	-	++	+++	+++
**Kidney**							
Inflammatory cell infiltration	++	++	++	+++	+++	+++	+++
Dilated blood vessels	-	-	-/+	++	++	++	++
Fibrosis	-/+	-	-	-	-	++	++
Eosinophilic material	-	-	-/+	++	++	-	-
Cysts	++	-	-	-	-/+	-	-
**Spleen**							
Clusters of megakaryocytes	-/+	-/+	-	-/+	++	-/+	++
Macrophages	-	-/+	-	-	+++	-/+	-

B

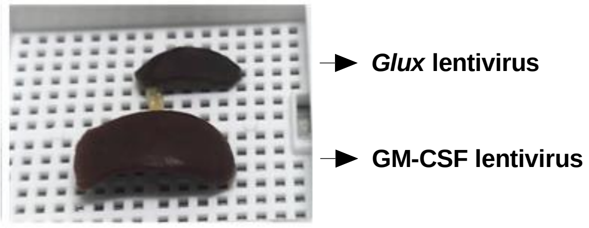

C

	Mice number (n)	Enlarged Spleen (%)
**PAP**	1/10	10
**Glux**	5/45	11
**1e7**	1/12	8
**5e7**	4/11	36
**25e7**	4/11	36
**92e7**	4/6	67

Lungs of GM-CSF knockout mice were treated at increasing doses of the rSIV.F/HN-mGM-CSF (1e7-92e7 TU/mouse) lentivirus. Histopathology was compared to wildtype mice (WT), untreated knockout mice (PAP) and knockout mice treated with a control vector (*Glux*, 24e7 TU/mouse) two months after treatment (n=3-6 mice/group). **(A)** Analysis was performed

blinded and scored using a semi-quantitative scoring system. Scores defined as: - absent, -/+ equivocal, ++ mild, +++ moderate, ++++ severe. PAM=pulmonary alveolar macrophage. Spleen weight was measured and the percentage of animals with enlarged spleen (> 0.2 g) was calculated per treatment group. **(B)** Representative image of an enlarged spleen after treatment with the *Glux* or the GM-CSF lentivirus. **(C)** Proportion of mice with an enlarged spleen.

Supplementary Table 1: Time-related histopathological changes after pulmonary administration of rSIV.F/HN-mGM-CSF to GM-CSF knockout mice

	**1 week**		**1-2 months**		**6 months**		**9 months**	
**Lung**	***Glux***	**GM-CSF**	***Glux***	**GM-CSF**	***Glux***	**GM-CSF**	***Glux***	**GM-CSF**
Distorted architecture	-	-	-	-	-	-	-	-
Inflammatory cell infiltration	++	+++	+++	+++	+++	+++	-	++++
Alveolar wall thickness	-	-/+	-	-/+	-	-	-	++++
PAM alveoli	-/+	+++	-	+++	-/+	++++	+++	++++
PAM bronchi	-	+++	-	-/+	-	+++	++	++++
Neutrophils bronchi	-	-	-	-	-	++	-	++++
Consolidation	-	+++	-	-	-	-/+	-	++++
Giant cells	-	-	-	-	-	-/+	-	++
Eosinophilic material	-	-	-	-	-/+	-	-	-/+
Oedema	-	-/+	-	-	-	-	-	-/+
**Kidney**								
Inflammatory cell infiltration	-	-/+	-/+	++	-/+	++	++	+++
Dilated blood vessels	-	-	++	-	-/+	-	-	+++
Fibrosis	-	-	-	-	-	-	-	-
Epithelial loss tubules	-	-	-	-	-	-	-	-
Eosinophilic material	-	-	-	-	-	-	-	-
Cysts	-	-	-	-	-	-	-	-

Lungs of GM-CSF knockout mice were treated with rSIV.F/HN-mGM-CSF or the *Glux* control lentivirus (1e7 TU/mouse) (n=3-10 mice/group). Histopathology was compared between GM-CSF and *Glux* treated mice at 1 week to 9 months after treatment. Analysis was performed blinded and scored using a semi-quantitative scoring system. Scores defined as: - absent, -/+ equivocal, ++ mild, +++ moderate, ++++ severe. PAM=pulmonary alveolar macrophage.
